# Soil quality parameters vis-a-vis growth and yield attributes of sugarcane as influenced by integration of microbial consortium with NPK fertilizers

**DOI:** 10.1038/s41598-020-75829-5

**Published:** 2020-11-05

**Authors:** S. K. Shukla, Lalan Sharma, V. P. Jaiswal, A. D. Pathak, Raghvendra Tiwari, S. K. Awasthi, Asha Gaur

**Affiliations:** ICAR-Indian Institute of Sugarcane Research, P.O. Dilkusha, Lucknow, 226002 India

**Keywords:** Microbiology, Plant sciences

## Abstract

Intensive agriculture involving high crop intensity, unavailability of organics, and higher use of straight fertilizers causes imbalanced use and deficiencies of several other macro and micronutrients. Nutrients supply through the integration of microbial consortium containing *Gluconacetobater diazotrophicus, Trichoderma harzianum, and Pseudomonas fluorescens* can reduce the requirements on the one hand and can also increase the response of chemical fertilizers. Thus we had planned the present experiment with the objectives (i) to determine the effect of integrated application of microbial consortium (MC) and NPK fertilizer on soil quality parameters and crop growth and yield attributes and (ii) to assess the effect of integration on agronomic efficiency of N, P and K and find out the possibilities for reduction in applied doses of NPK, if any. Five treatments viz., T_1_; N_0_P_0_K_0_; T_2_: N_75_P_13_K_25_; T_3_: N_150_P_26_K_50_; T_4_: N_75_P_13_K_25_ + microbial consortium and T_5_: N_150_P_26_K_50_ + microbial consortium containing new strains of *Trichoderma harzianum*, *Gluconcetobacter diazotrophicus*, and *Pseudomonas fluorescens* (CFU 10^9–10^ per ml liquid culture) were evaluated under four replications in a randomized block design (RBD). Experimental results indicated that integrating microbial consortium and NPK fertilizers' application proved effective in improving soil organic carbon, soil microbial population, microbial biomass carbon, microbial biomass nitrogen, and soil respiration. Integrated use of microbial consortium with NPK also improved the cation exchange capacity of soil and roots. However, the growth and yield attributes, nutrients uptake, sugarcane, and sugar yields also revealed a positive effect of microbial consortium's integrated application with NPK. The integration of MC and NPK also improved the agronomic efficiency of applied nutrients (NPK). Reduction of 50% NPK with these microbial consortia (*Trichoderma harzianum*, *Gluconcetobacter diazotrophicus*, and *Pseudomonas fluorescens*) was found better than the application of full NPK through chemical fertilizers. Thus application of N_150_P_26_K_50_ with microbial consortium can sustain soil fertility besides improving sugarcane and sugar yields in subtropical Indian conditions.

## Introduction

Microbes' role in modern agriculture is increasing due to declining soil organic carbon content^1^ and decreasing partial factor productivity. Sustainability of the crop production system is a vital component to feed the growing population^2^. Intensive agriculture involving high crop intensity, unavailability of organics, and higher use of straight fertilizers causes imbalanced use and deficiencies of several other macro and micronutrients^3^. The higher use of chemical fertilizers is also affecting NO_3_^_^ pollution in soil water^4^ and CH_4_ and NO_2_ emission in the environment causing global warming besides adversely affecting soil chemical and biological properties^5^. Sugarcane crop-producing 100 tonnes cane yield per ha removes about 208 kg N, 53 kg P, and 280 kg K^6^. The requirement of nutrients varies with planting seasons, growing regions, and nutrient status availability in fields. Sugarcane is a heavy feeder crop supplied with 150–350 kg N; 60–100 kg P_2_O_5_, and 60–120 kg K_2_O ha^-1^ annually depending upon planting time and growing regions of the country^6^.

Soil microbes and their functions related to C transformation and turnover are influenced by N application^7^. Microbes also play an important role in decomposition processes, including lignin and cellulose degradation and soil carbon turn over^8–10^. *Gluconacetobacter diazotrophicus* is an endophytic bacteria and survives under anaerobic conditions in plant roots, rhizomes, stalk, and leaves of sugarcane^11^. Flavonoids exuded by the crop sources serve as signals to enter, colonize, and fix atmospheric dinitrogen by reducing into ammonia^12^. Several experiments in India and other countries revealed saving of 25–50% N through the application of *Gluconacetobacter* bacteria in sugarcane^13,14^. *Pseudomonas fluorescens* acts as P solubilizer and improves solubility of P by converting PO_4_^–3^ to HPO_4_^–2^ and H_2_PO_4_^–1^ ^15,16^. Increasing P content in sugarcane juice is an utmost important task for better crystallization of sugar during crushing. Usually > 300 ppm phosphate concentration in sugarcane juice provides better crystallization of sugar, which improves sugar recovery in mill^17^. The phosphorus uptake in cane and its diversion in juice could be increased through efficient use of unavailable P in soil. *Trichoderma* fungus controls soil-borne pathogens^18,19^ and also makes the rhizospheric environment congenial for the growth of sugarcane crops through the release of cell wall degrading enzymes, secondary metabolites, etc.^20^. Thus, *Trichoderma* also acts as bio decomposer and having the potential to increase soil organic carbon besides improving crop growth and yields^21–23^.

Various experiments have been conducted to determine the effect of the above microorganisms on the growth and yield of sugarcane. However, an integrated application of these microbes with chemical fertilizers has great scope keeping in view the reduction of macronutrients supplied through chemical fertilizers and improving the nutrient use efficiencies besides increasing crop yields. *Gluconacetobacter diazotrophicus* can reduce the N level. However, up to 50% requirement of P can also be supplemented/substituted with P solubilizers with chemical fertilizers^24–26^ *Trichoderma* also acts as a growth promoter and, if augmented with chemical fertilizer, increased sugarcane yield and soil quality parameters^21,23^.

Thus the individual effect of these microbes on the growth and yield of crops has been reported by several researchers. Work has also been done on the aspect of containing pathogen by *Trichoderma* spp^19,22^. The reports on P solubilizer also revealed the performance of various *Pseudomonas* or *Bacillus spp* on P solubilizing capacity and its effect on reducing P level in various crops^27,28^. The interactions of diverse microbes in soil play the most crucial role in optimizing the rhizospheric environment for better plant growth. The performance of these microorganisms varies in different ways. Complementarities between *Trichoderma, Gluconacetobacter, and Pseudomonas* spp is an essential issue for efficient utilization of these microbes and assessing their effect on soil quality parameters, crop growth, and yields as well. The scientific information on reducing NPK through the combined use of these microorganisms is lacking. It holds great promise for sustaining soil fertility and crop productivity in the sugarcane-based system. The variations in these microbes' effectivity have also been observed, and potential strains of these microbes have been identified at the India Institute of Sugarcane Research, Lucknow. These microbes have also been deposited in GenBank, and accession numbers had also been provided. Therefore, a need was felt for a systematic study on assessing microbial consortium, its effect on soil quality parameters, sugarcane growth, yields, and possibilities to reduce NPK level to sugarcane crop.

Thus present experiment was planned, keeping the following objectives in view viz., (i) to determine the effect of integrated application of microbial consortium (MC) and NPK fertilizer on soil chemical and biological parameters. (ii) to assess the impact of integrated application of MC and NPK fertilizer on dry matter accumulation, nutrients uptake pattern, yield attributes, and sugar yield (iii) to assess the effect of integration on nutrients use efficiencies (agronomic efficiencies of N, P and K) exploring the possibilities of reduction in applied doses of NPK, if any.

## Materials and methods

### The Experimental site, climate, and soil

**Figure 1:**
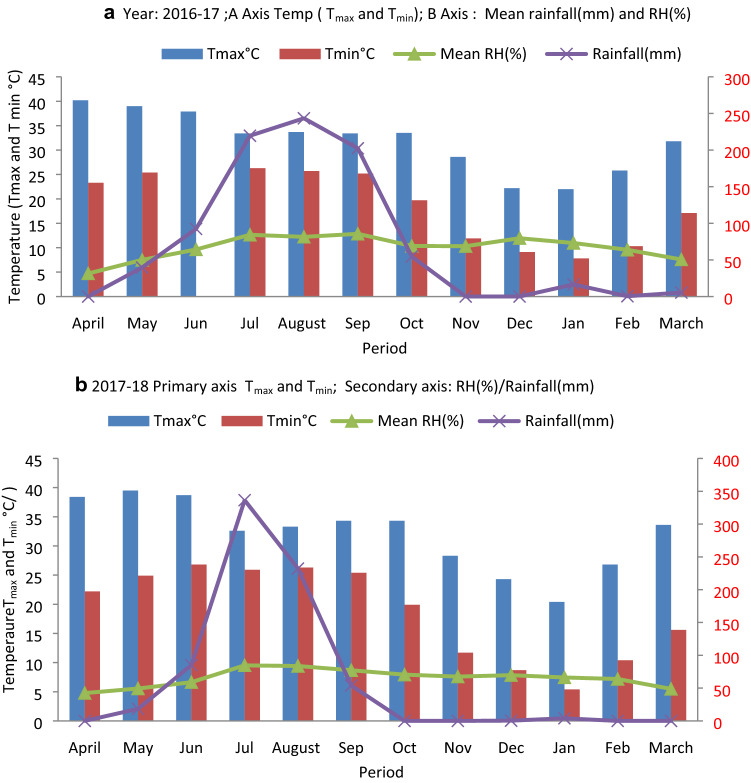
(**a**) Climatological parameters (Temp/rainfall/relative humidity) during the first cropping season (2016–2017). (**b**) Climatological parameters (Temp/rainfall/relative humidity) during the first cropping season (2017–2018).

A field experiment was conducted during 2016–18 at the ICAR-Indian Institute of Sugarcane Research, Lucknow, located at 26.50° North and 80.50° East at an elevation of 123 m above sea level. The climate of the area comes under the category of subtropical (semi-arid region) having dry, hot summer (April–June), rainy season (July–September), winter (November–January). Autumn (October) and spring (February–March) are moderately cooler with moderate temperatures and humidity and are considered best for the planting sugarcane. The data on meteorological parameters viz., temperature, rainfall, and humidity have been presented through Fig. 1a,b. The soil of the experimental field was loam (45% sand, 35% silt, and 20% clay) having pH 7.60, Electrical conductivity (0.26 ds m^−1^), soil organic carbon (19.75 Mg ha^−1^), available N (304.2 kg Na ha^−1^), available P (16.32 kg ha^−1^) and available K (376.3 kg ha^−1^). Initial soil sampling from five spots in the experimental field was done through a Core Sampler of 8 cm diameter in 0–15 cm depth to determine physical, chemical, and biological properties. The initial bulk density of soil at 0–15 cm depth was recorded as 1.40 Mg m^−3^. Soil organic carbon content (Mg ha^−1^) was calculated by the formula given below.

$${\text{SOC }}\left( {{\text{Mg ha}}^{{ - {1}}} } \right) = {\text{ Soil organic carbon }}\left( {{\text{g kg}}^{{ - {1}}} {\text{soil}}} \right) \, \times { 2}.{2}0 \, \times {\text{ bulk density }}\left( {{\text{Mg M}}^{{ - {3}}} } \right)$$

Initial soil microbial population viz., bacteria (48 × 10^5^ CFU per g soil), fungi (2.50 × 10^3^ CFU per g soil), and actinomycetes (2.10 × 10^3^ per g soil) in 0–15 cm depth was determined. Other soil microbial activities viz., soil microbial biomass carbon (624.4 mg CO_2_-C/kg soil/day), soil microbial biomass nitrogen (12.4 mg NH_3_-N/kg soil/day), and soil respiration (204 mg CO_2_-C/kg soil/day) were also determined before experimentation. The initial cation exchange capacity of the soil was determined as 42.8 Meq/100 g soil.

### Preparation and application of microbial consortium of *P. fluorescens*, *G. diazotrophicus* and *T. harzianum*

The pure culture of *Pseudomonas fluorescens* strain PSB28 (GenBank Acc. No. MH817418), *Gluconacetobacter diazotrophicus* strain NB73(GenBank Acc. No. MT012287), and *Trichoderma harzianum* strain T6 (GenBank Acc. No. MH151122) were isolated at the Soil–Plant–Water Analysis Lab of ICAR-Indian Institute of Sugarcane Research, Lucknow. The bacterial culture was prepared from a single colony of *P. fluorescens* and *G. diazotrophicus* in 1000 ml of sterilized Pikovskaya broth^29^ and LGI broth^30^. The inoculated bacterial culture flask was incubated at 30 °C for five days. After five days of incubating bacterial culture at 30 °C, the bacterial culture was counted for colony-forming units (CFUs). It was recorded that bacterial culture broth of *P. fluorescens* strain PSB28 had approximately 4.5 × 10^9^ cells/ml and broth of *G. diazotrophicus* strain NB73 had about 3.9 × 10^9^ cells/ml, respectively. The mass inoculum of *T. harzianum* strain T6 was prepared by preparing starter culture on potato dextrose broth medium and incubated at 30 °C for 20 days. For good sporulation of *T. harzianum* culture, every third day’s proper inoculum shaking and mixing was done. The prepared *T. harzianum* (*Th*) based broth culture inoculum containing approximately 5 × 10^9^ *Th* fungal counts/ml. The broth-based inoculum of these microbial cultures (bacterial and fungal) at equal proportion were mixed and diluted with sterilized distilled water. The microbial inoculum carrying sterilized water was sprayed on sugarcane setts in furrows before soil covering.

### Assay of microbial population determination

For microbial population determination before experimentation, the spread plate method was used. The spread plate method provides information on total counts/viable cells. The broth multiplied bacterial (*Pseudomonas fluorescens* and *Gluconacetobacter diazotrophicus)* and fungal (*Trichoderma harzianum*) samples (0.1 ml) of suitably diluted suspensions were surface plated on to nutrients agar and potato dextrose agar plates, respectively. All the platings were made in triplicate to minimize errors on the microbial population count. The microbial colonies were counted after incubation for 48 h at 30 °C. The presence of single type colonies on the surface plated petri dish indicates the pure culture of microbes. It was recorded that bacterial culture broth of *Pseudomonas fluorescens* and *Gluconacetobacter diazotrophicus* has approximately 4.5 × 10^9^ and 3.9 × 10^9^ cells/ml, respectively. The prepared *Trichoderma harzianum* based broth culture inoculums were plated and counted about 5 × 10^9^ *Th* fungal counts/ml. The broth-based inoculums of these microbial cultures (bacterial and fungal) were mixed in equal proportion at the application time. The quantified appropriate microbial inoculums carrying sterilized water was sprayed on sugarcane setts in furrows before soil covering. The formula adopted for microbial population determination was – Colony-forming units/ml = (no. of colonies × dilution factor)/volume of culture plate.

### Crop culture

The experimental field was prepared for planting after one plowing through a moldboard plough and two ploughings through tractor operated disc harrow/cultivator. After planking, the area was leveled, and the layout was done. Deep furrows (15–20 cm) were opened at 75 cm row spacing, and three bud setts of sugarcane (Cv. Co0238) were used for planting. A basal dose of 1/3rd N and full P and K were applied as per treatments. Basal application of 1/3rd N and full P and K (as per treatments) was used through urea, diammonium phosphate, and muriate of potash (KCl). Three bud setts were placed manually in furrows keeping end to end adjustment and pressed in soil. About 53,300 three bud setts were required for planting of one ha area. After manually placing, setts were covered through soil not more than 2–3 inches on setts. Topdressing of the remaining 2/3rd N was done during the tillering stage (April–June) in two equal splits. Planting was done on 16th February 2016 and on 18th February 2017 during the first and second cropping seasons. Another package of practices recommended for raising sugarcane crop in the region was followed. The sugarcane crop was harvested on 28th December 2016 and 29th December 2017 during the first and second cropping seasons.

### Treatments

Five treatments, viz., T_1_; N_0_P_0_K_0_; T_2_: N_75_P_13_K_25_; T_3_: N_150_P_26_K_50_; T_4_: N_75_P_13_K_25_ + microbial consortium and T_5_: N_150_P_26_K_50_ + microbial consortium containing *Trichoderma harzianum*, *Gluconcetobacter diazotrophicus*, and *Pseudomonas fluorescens* (CFU 10^9–10^ per ml liquid culture) were applied. These treatments were evaluated under four replications in a randomized block design (RBD). Planting of eight rows (at 75 cm spacing) of 6-m length was done in each plot. Thus minimum plot size was kept at 36 m^2^. Destructive sampling was done through the 2^nd^ row of each plot. Net sugarcane yield at the harvest was worked out based on four rows leaving border rows and sample rows in each plot.

### Soil studies

Various soil quality parameters were determined at different growth stages (tillering, grand growth, and harvest). Sampling for tillering, grand growth, and harvest stages was done in June, September, and December, respectively, during both the seasons. The bulk density of soil was determined by the Core Sampler method (Blake, 1965). Soil texture (sand, silt, and clay percent) was determined through the Bouyoucos hydrometer method^31^. Soil pH and electrical conductivity (EC) were determined in 1:2.5 soil–water suspensions by pH Meter and Electrical conductivity meter, respectively. Available N in soil (alkaline permanganate method^32^), available P (Olsen P ascorbic acid method^33^), and available K^34^ were determined. The determination of total soil fungi was done through the Rose Bengal medium method^35^. However, total bacteria through plate count agar method^36^ and actinomycetes through Kenknight method^37^. At different growth stages (tillering, grand growth, and harvest), soil microbial biomass carbon and soil microbial biomass nitrogen were determined through the chloroform fumigation-incubation method^38^. Soil respiration was also determined through unfumigated soil incubation method^38^. The soil's cation exchange capacity was determined through the method prescribed by Chapman (1965) and calculated by the following formula.

$${\text{CEC }}\;{\text{meq}}/{1}00\;{\text{g}}\;{\text{ soil}} = \frac{{{\text{X}}^{**} - {\text{ Y}}^{*} \times { 1}00 \, \times {\text{ Normality }}\;{\text{of}}\;{\text{ titrant}}}}{{{\text{soil wt }}\left( {\text{g}} \right)}}$$

*Volume of acid used for titrating blank.

**Volume of acid used for titrating the sample.

### Crop studies

At various growth stages, ten sugarcane plants were randomly harvested in each plot. Tillers were partitioned in leaf and stalk after harvesting. These samples were oven-dried at 65 °C for 72 h after proper sun drying. Tillers counting at different growth stages were done and expressed in thousand per ha. Thus total dry matter accumulation per unit area of the above-ground part (leaf and stalk) was worked out. During the sampling, the plants were cut through a sharp knife blade hand tool (*Ganasha*) from the ground level. After drying in the shed, these samples were kept in the hot air oven at 60 °C to a constant weight. Total N content in leaf and stalk of sugarcane was determined by the Kjeldahl method. Phosphorous in the plant sample was also determined by Vanadomolybdo phosphoric yellow colour method^39^. Plant potassium content was determined by the Flame photometer method. The NPK contents in stalk and leaf at all the growth stages were determined and presented as kg ha^-1^ after multiplying their contents with dry matter accumulation at respective stages.

Ten canes were randomly selected from each plot for determination of juice quality parameters. Yield attributes viz., individual length, diameter, and weight were recorded after sampling. Quality parameters of cane juice (°brix, pol percent, and purity) were determined through Auto Pol. Commercial cane sugar (%) was determined through the following formula^40^.

$${\text{CCS }}\left( \% \right) = \left\{ {{\text{S}} - \left( {{\text{B}} - {\text{S}}} \right) \, \times \, 0.{4}} \right\} \, \times \, 0.{73}$$

where S is the Sucrose percent juice, and B is the °Brix.

Commercial cane sugar (CCS) or sugar yield per ha was estimated by multiplying CCS (%) with sugarcane yield.

$${\text{Sugar yield}}/{\text{CCS }}\left( {{\text{t}}/{\text{ha}}} \right) \, = {\text{ CCS }}\left( \% \right) \, \times {\text{ Sugarcane yield }}\left( {{\text{t ha}}^{{ - {1}}} } \right) \, /{ 1}00$$

Agronomic efficiency of N, P, and K was also worked out as per the following formula^41^.

$${\text{AE}} = {\text{Crop }}\;{\text{yield }}\left( {{\text{kg}}} \right)/{\text{N}},{\text{ P or K}}\;{\text{ applied}}$$

Cation exchange capacity of roots was determined through the method given by Chamuah^42^.

$${\text{CEC}}\;{\text{ meq}}/{1}00 \, \;{\text{g }}\;{\text{root }} = \frac{{{\text{X}}^{**} - {\text{ Y}}^{*} \times { 1}00 \, \times {\text{ Normality}}\;{\text{ of}}\;{\text{ titrant}}}}{{{\text{Root }}\;{\text{dry }}\;{\text{wt }}\left( {\text{g}} \right)}}$$

*Volume of acid used for titrating blank.

** Volume of acid used for titrating the sample.

### Statistical analysis

The data on various soil and plant characters recorded were statistically analyzed in RBD. Pooled analysis of two years crop data was done^43^, and mean results have been presented for analysis on scientific principles, discussions, and conclusions. Statistical analysis was done through a software program on RBD^44^, and the critical difference (CD) at a 5% probability level was determined to assess the significant differences among various treatments.

## Results

### Soil organic carbon (SOC), soil microbial biomass carbon (SMBC), soil microbial biomass nitrogen (SMBN), and soil respiration (SR)

The data on soil organic carbon (SOC) during various crop growth stages (Table 1) indicated inoculation of *Trichoderma harzianum, Pseudomonas fluorescens*, and *Gluconacetobacter diazotrophicus* (microbial consortium-MC) with recommended dose of NPK increased SOC up to grand growth stage. However, after the grand growth stage, a marginal decrease in SOC was recorded with all the treatments, including NPK + MC. Despite this, the application of MC with NPK increased SOC by 17.86% (21.25 Mg ha^−1^) compared to NoPoKo and 10.05% compared to N_150_P_26_K_50_. Thus inoculation of MC with NPK fertilizers increased SOC as compared to inorganic fertilizers. Reduction of NPK to 50% with MC showed higher SOC than full NPK fertilization (N_150_P_26_K_50_).

**Table 1:** Soil organic carbon and available nutrients status in soil during crop growth at various stages.

Treatment	SOC (Mg ha^−1^)			SMBC(CO_2_-C mg kg^−1^ soil day^−1^			SMBN(NH_3_-N mg kg^−1^ soil day^−1^)			Soil respiration(CO_2_-C mg kg^−1^ soilday^−1^)		
	Tillering	Grand growth	Harvest	Tillering	Grand growth	Harvest	Tillering	Grand growth	Harvest	Tillering	Grand growth	Harvest
T_1_: N_0_P_0_K_o_	19.96	21.25	18.03	574.5	1533.3	831.1	11.4	14.7	12.4	198.0	242.0	165.0
T_2_:N_75_P_13_K_25_	20.44	23.18	19.64	634.5	1757.8	855.6	13.7	15.2	14.8	209.0	297.0	176.0
T_3_:N_150_P_26_K_50_	21.73	22.85	19.31	684.5	1906.7	880.0	15.6	17.4	16.0	242.0	319.0	198.0
T_4_:N_75_P_13_K_25_ + MC	21.62	22.64	20.13	712.4	1967.4	912.3	15.9	17.9	16.3	263.4	325.3	214.5
T_5_:N_150_P_26_K_50_ + MC	21.57	23.50	21.25	855.6	2102.2	953.3	17.1	19.6	17.3	275.0	341.0	264.0
CD for years	0.529	0.516	0.577	116.8	224.3	105.9	1.70	2.06	1.761	23.8	33.6	23.3
CD for treatments	1.20	1.32	1.25	67.35	134.45	67.34	0.87	0.92	0.78	14.39	16.45	13.45
CD for years × treatments	NS	NS	NS	NS	NS	NS	NS	NS	NS	NS	NS	NS
Initial	19.75			624.6			12.4			204		

Soil microbial biomass carbon (Table 1) also increased at the grand growth stage. The mean increase in SMBC at the grand growth stage was recorded by 165.5% (1825 mg CO_2_-C/g soil/day) compared to the tillering stage. However, SMBC between the grand growth phase to the harvest stage was declined (880 mg CO_2_-C/kg soil/day. Inoculation of MC with NPK increased SMBC at all the growth stages. The lowest SMBC was recorded with N_0_P_0_K_0_ (control). Inoculation of MC with recommended NPK showed 20.0% higher SMBC at the tillering stage (855.6 CO_2_-C kg soil/day) over the recommended dose of NPK. However, SMBC at the grand growth phase increased by 137% over control (N_0_P_0_K_0_).

Soil microbial biomass nitrogen also increased during the grand growth stage (16.73 mg NH_3_-N/kg soil/day), and marginal decrease (1.6 mg NH_3_-N/kg soil/day) was recorded during the grand growth to maturity phase. The inoculation of the microbial consortium also increased SMBN at all the growth stages. Reduction of 50% NPK and integration of microbial consortium increased SMBN compared to recommended NPK (N_150_P_26_K_50_) at all the growth stages. However, with recommended NPK, the application of MC further improved the soil microbial biomass nitrogen and increased the response.

Soil respiration is an indication of the microbial population in the rhizosphere. Mean soil respiration (231.00 mg CO_2_-C/kg soil/day) was higher during the tillering stage than the harvest stage. However, the most increased soil respiration (299.8 mg CO_2_-C/kg soil/day) was also recorded during the grand growth phase. The increasing dose of NPK favoured soil respiration at all the growth stages. However, the application of MC with recommended NPK further improved soil respiration by 13.645 to 33.33% at various growth stages than recommended NPK. At the harvest stage, soil respiration with recommended NPK + MC was increased by 60% as compared to N_0_P_0_K_0_ and about 33.3% over N_150_P_26_K_50_.

Integration of MC with reduced NPK also improved soil respiration (SR) and proved superiority over recommended NPK. Thus SOC, SMBC, SMBN, and soil respiration showed improvement due to inoculation of microbial consortium along with NPK fertilizers. Reducing the NPK level to N_75_P_13_K_25_ also improved soil chemical and biological activities with microbial consortium integration. However, the integration of MC with recommended NPK further increased the soil quality parameters and proved superiority to integration with a reduced level of NPK.

### Available nutrients (NPK) in soil

Integration of microbial consortium enhanced nutrients availability at all the growth stages (Table 2). Available N during all the growth stages ranged from 308.0 to 348.9 kg ha^−1^. The highest available N content was recorded during the grand growth stage. Increasing doses of NPK also increased the available N content of soil. Available N increased during the grand growth phase as compared to the tillering stage, but after completion of the grand growth phase, it declined during the maturity of the crop (Table 2). About 40 kg N ha^−1^ in soil was decreased during the grand growth to maturity phase. At the lower doses of NPK, approximately 14.28% available N could be increased at the grand growth stage with the integration of microbial consortia. However, at the recommended level of NPK, it could be improved by 9.16% during the same phase. The soil's highest P content was recorded during the tillering phase (20.25 kg P ha^−1^). It decreased to 17.45 kg ha^−1^ during the grand growth phase and further improved during maturity (18.16 kg ha^−1^). The inclusion of PSB in microbial consortium improved the available P content in soil during all the growth stages. Integration of microbial consortium improved P availability, and with 50% NPK showed higher P content than recommended NPK without MC. At the tillering stage, the application of microbial consortium also improved P content in soil by 16.38% and 7.47% with reduced NPK and recommended level, respectively. Potassium content in the soil was the highest (425.8 kg ha^−1^) during the tillering phase. However, it decreased to 387.7 kg ha^−1^ during the grand growth phase and further reached 343.8 kg ha^−1^ at the harvest stage. Inoculation of MC with a reduced level of NPK and at the recommended level improved 12.06% and 8.18% available K, at the tillering and grand growth stages, respectively. Sugarcane crops heavily remove potassium, and the application of K through inorganic fertilizer enhanced K availability in soil. The microbial consortium also improved K accumulation and ensured higher availability of sugarcane crop.

**Table 2:** Available nutrients status (NPK kg ha^−1^) in soil at various stages during crop growth.

Treatment	N			P			K		
	Tillering	Grand growth	Harvest	Tillering	Grand growth	Harvest	Tillering	Grand growth	Harvest
T_1_: N_0_P_0_K_o_	290.5	294.5	271.3	15.10	14.15	15.90	369.86	325.40	305.93
T_2_:N_75_P_13_K_25_	320.2	329.2	291.6	19.48	18.14	17.73	403.79	371.08	338.61
T_3_:N_150_P_26_K_50_	338.7	369.1	312.6	22.37	18.15	17.96	453.90	410.32	353.23
T_4_:N_75_P_13_K_25_ + MC	336.3	376.2	332.3	22.67	18.32	18.40	452.5	396.3	367.2
T_5_:N_150_P_26_K_50_ + MC	351.2	402.9	356.6	24.04	19.36	21.03	475.71	443.89	377.60
CD for years	39.3	39.9	30.194	2.500	2.112	2.220	51.90	46.025	42.19
CD for treatments	16.45	22.34	18.34	1.27	1.12	1.20	22.56	24.35	27.39
CD for years × treatments	NS	NS	NS	NS	NS	NS	NS	NS	NS

### Cation exchange capacity of root and soil

Cation exchange capacity of soil represents the capacity to hold the exchangeable cations on clay complex. The improved cation exchange capacity of soil and roots showed the active ions adsorption and governed nutrients availability to crop. Cation exchange capacity of roots was worked out on 100 g dry weight of roots during different growth stages (Table 3). The mean cation exchange capacity (CEC) during the tillering stage was the highest (47.33 meq per 100 g dry weight of roots) as compared to grand growth (42.08 meq per 100 g dry weight of roots) and harvest stage (37.5 meq per 100 g dry weight of roots). Increasing the NPK level and integration of MC improved the cation exchange capacity of roots at all the growth stages because of the higher number of cations available. Microbial consortium affected 13.45% and 10.64% improvement with reduced NPK and recommended NPK level, respectively. Cation exchange capacity of soil also followed a similar pattern as root CEC. However, the CEC of soil increased during the grand growth phase (5.44 meq/100 g soil) and declined during maturity because of a lower level of nutrients. Integration of MC with recommended NPK improved soil CEC by 17.75% at the harvest stage compared to control (N_0_P_0_K_0_). However, improvements during the tillering and grand growth phase were recorded as 28.92% and 25.58%, respectively, with N_150_P_26_K_50_ + MC over N_0_P_0_K_0_. Application of MC with NPK showed the highest CEC of soil (5.94 meq/100 g soil) during the grand growth phase of the sugarcane crop. Microbial consortium affected 5.83% and 7.57% improvement with reduced NPK and recommended level, respectively.

**Table 3:** Cation exchange capacity of root and soil during crop growth.

Treatment	CEC root Meq per 100 g dry wt of roots			CEC soil meq per100 g dry wt of soil		
	Tillering	Grand growth	Harvest	Tillering	Grand growth	Harvest
T_1_: N_0_P_0_K_o_	39.34	37.78	34.43	4.46	4.73	3.38
T_2_:N_75_P_13_K_25_	45.30	39.02	36.03	5.36	5.47	3.60
T_3_:N_150_P_26_K_50_	48.57	43.44	38.95	5.38	5.60	3.70
T_4_:N_75_P_13_K_25_ + MC	47.27	44.27	38.12	5.42	5.73	3.81
T_5_:N_150_P_26_K_50_ + MC	56.09	48.06	40.69	5.75	5.94	3.98
CD for years	5.70	4.673	4.53	0.645	0.674	0.447
CD for Treatments	2.76	2.45	2.82	0.32	0.34	0.28
CD for years × treatments	NS	NS	NS	NS	NS	NS

### Soil microbial population (bacteria, fungi, and actinomycetes)

**Figure 2:**
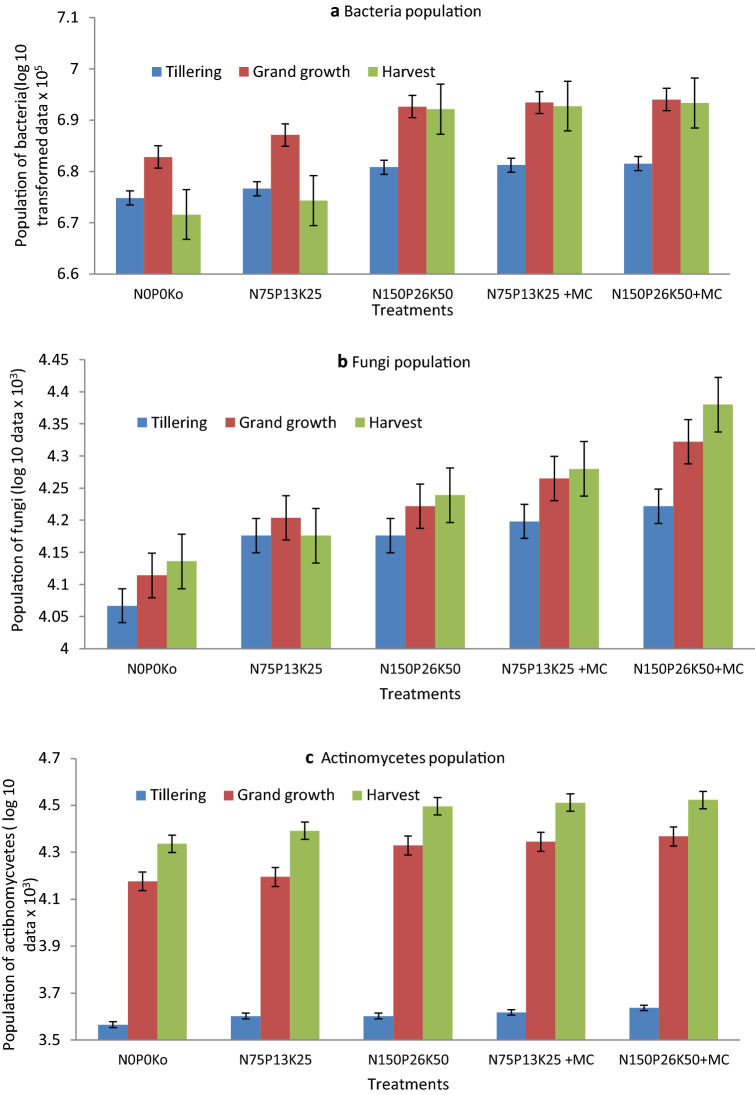
(**a**) Dry matter accumulation and its partitioning in leaf and stalk during the tillering stage as influenced by various treatments. (**b**) Dry matter accumulation and its partitioning in leaf and stalk during the grand growth stage as influenced by various treatments. (**c**) Dry matter accumulation and its partitioning in leaf and stalk at the harvest stage as influenced by various treatments.

The data on soil microbial population (bacteria, fungi, and actinomycetes) during various growth phases of sugarcane have been presented through Fig. 2a–c. The logarithmic transformation of data revealed that the MC's integration with NPK improved the population of all microbes at all the growth stages. Mean bacterial numbers during all the growth stages ranged from 61.0 × 10^5^ CFU per g soil to 78.2 × 10^5^ CFU per g soil. However, the lower population of fungi was recorded (14.6 × 10^3^ CFU per g soil to 17.5 × 10^3^ CFU per g soil) as compared to bacteria. The population of actinomycetes was recorded at the lowest (4.0 × 10^3^ CFU per g soil) during the tillering stage. The population of all the microbes improved due to the application of NPK and MC. Reducing NPK dose and inclusion of microbial consortium increased microbial population as compared to recommended NPK. During the grand growth stage, integration of MC improved 14.97% bacterial population at the reduced NPK level. However, the population could be increased by 52.23% at the harvest stage. Inoculation of MC also recorded a higher increase (38.49%) in population of fungi at the harvest stage with the recommended level of NPK compared to a reduced level. The higher population of all the microbes was recorded during the grand growth phase except actinomycetes. The population of actinomycetes was recorded at the highest level during the maturity phase. Recommended NPK + MC improved bacterial population in the range of 16.66% and 64.75% during the tillering and harvest stage, respectively, as compared to control (N_0_P_0_K_0_). The fungi population also improved in the range of 42.84% and 75.57%, respectively, and the corresponding increase for actinomycetes during the tillering and harvest stage was found as 17.98% and 53.81%, respectively, over the control (N_0_P_0_K_0_). Inoculation of MC at the reduced level of NPK also improved the population of actinomycetes by 41.42% compared to the recommended NPK (9.38% increase) during the grand growth stage.

### Tiller population and partitioning of dry matter accumulation

The periodic tiller population increased from 60,800 ha^−1^ in March to 98,700 ha^−1^ in September (Table 4). Tiller number increased till June during high temperature and low humidity and reached at the highest level (166,700 ha^−1^). After that, the tiller population declined due to their mortality in high temperature and high humid conditions prevailing during the monsoon/rainy season. In September, the mean tiller population reached to 98,700 ha^−1^. Application of NPK along with MC increased tillering in sugarcane during all the growth stages. An improvement of 38.45% to 58.55% was obtained with recommended NPK + MC compared to control (N_0_P_0_K_0_). However, the additional effect of MC on increasing tiller population in September was recorded as 11.24% higher than N_150_P_26_K_50_. Integration of MC with the reduced level of NPK increased tiller population by 13.48% in September. An increase in tiller population showed improved growth in the integrated application of inorganic fertilizer with MC.

**Table 4:** Periodic tiller population (000/ha) in sugarcane.

Treatment	March	April	May	June	July	August	Sep
T_1_: N_0_P_0_K_o_	48.6	68.5	90.4	134.5	112.4	94.3	74.4
T_2_:N_75_P_13_K_25_	52.75	88.00	104.63	164.70	139.71	113.17	96.23
T_3_:N_150_P_26_K_50_	67.61	96.56	138.70	181.13	144.78	124.29	106.04
T_4_:N_75_P_13_K_25_ + MC	65.32	99.23	156.23	183.4	149.2	128.4	109.2
T_5_:N_150_P_26_K_50_ + MC	74.39	104.23	167.10	186.46	155.62	133.16	117.96
CD for years	7.425	10.493	10.493	20.588	17.000	14.375	12.211
CD for treatments	4.65	6.38	7.62	8.20	7.34	6.84	6.72
CD for years × treatments	NS	NS	NS	NS	NS	NS	NS

**Figure 3:**
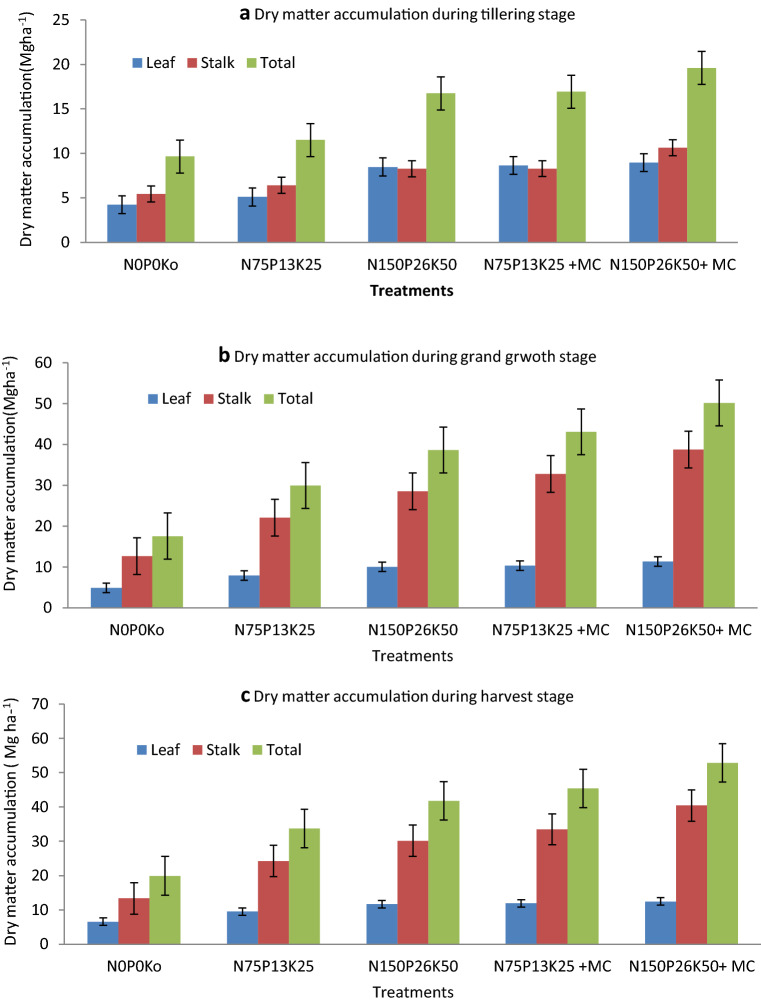
(**a**) Bacteria population in soil (log 10 × 10^5^ transformed data) as influenced by various treatments. (**b**) Fungi population in soil (log 10 × 10^3^ transformed data) as influenced by various treatments. (**c**) Actinomycetes population in soil (log 10 × 10^3^ transformed data) as influenced by various treatments.

Dry matter accumulation (DMA) at the different growth stages is partitioned into leaf and stalk and depicted through Fig. 3a–c. About 38.8% DMA of total dry matter (TDM) at the harvest (37.1 Mg ha^−1^) was recorded during tillering stage. The DMA increased at a faster rate and reached 34.1 Mg ha^−1^ (91.9% of TDM) at the grand growth phase. Partitioning in leaf and stalk showed that leaf contributed about 46.5% during the tillering phase-out of total biomass produced at tillering. Thus contribution of stalk was higher (53.5% of TDM at tillering). However, during the grand growth phase, the contribution of leaf reduced to 25.2%, and stalk increased to 74.8%. During the maturity stage also, the contribution of leaf and stalk to the TDM was recorded to 26.9% and 73.1%, respectively. Thus increasing biomass during the grand growth phase increased dry matter accumulation in stalk and showed diversion of photosynthates from source to sink. Tiller mortality was recorded during the grand growth phase, and elongation of cane diverted a higher share of biomass in stalk as compared to leaf. At all the growth stages, and improved dose of NPK accelerated biomass production in leaf and stalk. During the tillering phase, the application of NPK + MC recorded about 112.5% higher biomass accumulation as compared to control (N_0_P_0_K_0_) in leaf.

However, the increase in stalk was recorded at a level of 95.6% with the integration of NPK and MC over N_0_P_0_K_0_. Reduced NPK and inoculation of MC increased 34.51% TDM at the harvest stage as compared to N_75_P_13_K_25_. However, at the recommended NPK level, MC could increase the TDM by 26.65%. In both groups, improvement in dry stalk matter was greater than the total dry matter improvement. At the reduced NPK level, MC could improve dry stalk matter by 38.25% vis-a-vis 34.27% at the recommended NPK. It indicated the effect of microbial consortium in increasing biomass production. Reduction in NPK and integration with MC proved superiority over recommended NPK (N_150_P_26_K_50_), and it increased 11.16% higher biomass accumulation in stalk at the harvest stage as compared to recommended NPK.

### Root weight and volume

An increase in dry matter production resulted from vigorous root growth due to the higher absorption of nutrients from the soil. Fresh weight and Volume of active roots in 0–15 cm plough depth was determined at different growth stages (Table 5). The mean fresh weight of sugarcane roots increased to the grand growth phase (1.19 Mg ha^−1^) and decreased after that till harvest. Root volume also followed a similar trend as of root weight. During the tillering stage, it was about 1.03 M^3^ ha^−1^ and increased to1.5 M^3^ ha^−1^ during the grand growth phase and declined at 0.91 M^3^ ha^−1^ during maturity. Thus approximately 39.33% root volume diminished during the grand growth phase to the maturity phase. However, since the tillering phase, about 45.63% root volume increased up to the grand growth phase. It was because of the elongation phase of the crop, which favored an increase in root dry weight and root volume also. About 8.2% increase in mean root biomass was recorded during the grand growth phase as compared to the tillering stage. Thus it was observed that despite a lesser increase in fresh root weight, root volume increased at the higher rates during the grand growth phase. Improved fine roots (active) influenced root volume despite a lower rate of increase of root biomass. Integration of microbial consortium increased root weight and Volume at all the growth stages. Microbial consortium alone contributed higher root biomass in the range of 26.62% (at the harvest stage) to 62.87% (at tillering stage). However, root volume could be increased in the range of 26.62% and 79.34% at harvest and tillering stages, respectively. The effect of NPK on root biomass and Volume was upheld by the integration of microbial consortium with chemical fertilizer. Integration of MC with reduced NPK dose also showed superiority over recommended NPK. Increased root volume (40.19%) and root weight (18.78%) were recorded with NPK + MC as compared to the application of inorganic source (NPK) only.

**Table 5:** Fresh weight and volume of active roots during various stages of crop growth.

Treatment	Tillering		Grand growth		Harvest	
	Fresh root weight (Mg ha^−1^)	Root volume (M^3^ ha^−1^)	Fresh root weight (Mg ha^−1^)	Root volume (M^3^ ha^−1^)	Fresh root weight (Mg ha^−1^)	Root volume (M^3^ ha^−1^)
T_1_: N_0_P_0_K_o_	0.612	0.519	0.663	0.924	0.623	0.432
T_2_:N_75_P_13_K_25_	0.763	0.627	0.887	1.263	0.850	0.65
T_3_:N_150_P_26_K_50_	1.230	1.163	1.440	1.657	1.033	1.07
T_4_:N_75_P_13_K_25_ + MC	1.367	1.264	1.526	1.724	1.143	1.173
T_5_:N_150_P_26_K_50_ + MC	1.803	1.827	1.750	2.027	1.227	1.50
CD for years	0.122	0.130	0.157	0.175	0.100	0.122
CD for treatments	0.153	0.172	0.163	0.146	0.163	0.153
CD for years × treatments	NS	NS	NS	NS	NS	NS

### Nutrients (NPK) uptake

Nutrients uptake (NPK) presented in Table 6 indicated the positive influence of applying NPK and microbial consortium. About 31.2% N was accumulated in leaf at the harvest stage, (54.18 kg ha^−1^) and the remaining 69.8% (119.43 kg ha^−1^) in stalk. During the tillering phase, about 44.0% N accumulation was recorded in sugarcane compared to total N uptake at harvest. Partitioning of N in leaf and stalk showed a narrower margin (48.7% in leaf and 51.3% in stalk) during the tillering stage. However, during the grand growth phase, total N accumulation reached 167.44 kg ha^−1^, and leaf and stalk contributed 31.8% and 69.2%, respectively. The only marginal increase in N accumulation was recorded after the grand growth phase. Microbial inoculation with a reduced level of NPK brought forth 21.57% to 28.73% higher N accumulation during various growth stages. However, inoculation with the recommended NPK level also significantly influenced total N accumulation at all the stages (9.20% to 11.34% higher than recommended NPK). Thus, it was proved that by reducing NPK level and MC's inclusion, N accumulation could be improved. However, the response could be further enhanced with the recommended level of NPK + MC. About 15.44% higher N accumulation in stalk at the harvest stage could be analyzed due to the inclusion of microbial consortium with recommended NPK.

**Table 6:** Nutrients (NPK) uptake and partitioning in leaf and stalk at various growth stages of sugarcane.

Treatment	Tillering			Grand growth			Harvest		
	Leaf	Stalk	Total	Leaf	Stalk	Total	Leaf	Stalk	Total
	**N uptake kg ha**^**−1**^								
T_1_: N_0_P_0_K_o_	25.11	20.49	45.6	38.2	77.6	115.8	39	85	124
T_2_:N_75_P_13_K_25_	32.82	36.75	69.57	46.23	112.5	158.73	47.5	116.7	164.2
T_3_:N_150_P_26_K_50_	43.88	47.13	91.01	63.25	124.34	187.59	64.2	128	192.2
T_4_:N_75_P_13_K_25_ + MC	43.29	46.27	89.56	64.12	134.2	198.32	65.12	134.5	199.62
T_5_:N_150_P_26_K_50_ + MC	47.12	52.26	99.38	65.34	142.3	207.64	66	148	214
CD for years	4.638	4.894	9.558	6.720	14.311	21.014	6.821	14.834	21.65
CD for treatments	2.65	3.20	5.43	3.25	7.45	12.43	3.45	7.43	12.32
CD for years × treatments	NS	NS	NS	NS	NS	NS	NS	NS	NS
	**P uptake kg ha**^**−1**^								
T_1_: N_0_P_0_K_o_	3.10	3.48	6.58	6.92	15.38	22.3	7.9	16.2	24.1
T_2_:N_75_P_13_K_25_	3.34	3.81	7.15	7.91	19.86	27.77	10.63	20.17	30.8
T_3_:N_150_P_26_K_50_	5.73	6.03	11.76	9.32	25.36	34.68	11.85	24.35	36.2
T_4_:N_75_P_13_K_25_ + MC	5.89	6.23	12.12	9.76	27.45	37.21	11.45	26.76	38.21
T_5_:N_150_P_26_K_50_ + MC	6.96	7.03	13.99	10.77	30.77	41.54	11.24	31.26	42.5
CD for years	0.60	0.65	1.23	1.07	2.89	3.96	1.30	2.86	4.15
CD for treatments	0.65	0.58	0.63	0.72	2.30	3.20	0.62	1.80	3.30
CD for years × treatments	0.21	0.21	0.40	0.19	0.73	NS	0.20	0.69	0.87
	**K uptake kg ha**^**−1**^								
T_1_: N_0_P_0_K_o_	24.96	48.32	73.28	52.47	116.96	169.43	61.35	119.2	180.55
T_2_:N_75_P_13_K_25_	43.75	77.59	121.34	66.64	135.68	202.32	77.63	138.2	215.83
T_3_:N_150_P_26_K_50_	55.31	92.67	147.98	82.11	169.48	251.59	89.8	172.3	262.1
T_4_:N_75_P_13_K_25_ + MC	54.45	97.25	151.7	84.66	183.2	267.86	92.4	181.5	273.9
T_5_:N_150_P_26_K_50_ + MC	58.5	112.26	170.76	90.73	191.81	282.54	98.5	193.6	292.1
CD for years	5.73	10.37	16.10	9.12	15.38	28.41	10.16	19.50	29.64
CD for treatments	2.85	6.20	8.20	4.36	12.73	18.32	5.20	8.48	15.62
CD for years × treatments	NS	NS	NS	NS	NS	NS	NS	NS	NS

P uptake in leaf, stalk, and total accumulation at the harvest stage indicated the positive influence of NPK and the inclusion of microbial consortium. About 29.55% P of total uptake (33.40 kg ha^−1^) was accumulated during the tillering stage only. P accumulation increased to 31.57 kg ha^−1^ (94.5% of total) during the grand growth phase. Partitioning in leaf indicated 48.42%, 27.55%, and 31.16% during tillering, grand growth, and harvest stages, respectively, out of total P accumulated during respective stages. Thus mean P accumulation in stalk increased at greater rates after tillering. However, during the grand growth to the maturity phase, the marginal improvement was recorded. During tillering, P accumulation with full NPK and MC increased total P accumulation by 112.6% over the control (N_0_P_0_K_0_). However, the rate declined to 76.3% at the harvest stage. Microbial inoculation with reduced NPK increased higher P accumulation 24.06% higher P accumulation than application of N_75_P_13_K_25_. However, N_150_P_26_K_50_ + MC improved total P accumulation by 17.40% over N_150_P_26_K_50._ Reduced NPK with inoculation of MC improved about 9.90% higher P uptake in stalk than N_150_P_26_K_50_ (24.35 kg ha^−1^). It showed that microbial inoculation of MC gave a higher response with reduced NPK as compared to the recommended NPK level. Despite, with recommended NPK, integration of MC accumulated higher P as compared to sole NPK.

K uptake in stalk during the tillering stage was about 2.81 times higher (82.7 kg ha^−1^) than leaf (45.6 kg ha^−1^). Total K accumulation during the tillering stage was about 54% of the total accumulated K at the harvest. K accumulation during the grand growth phase was approximately 95.3% of total K at the harvest. The contribution of leaf during tillering, grand growth, and harvest stage was 35.54%, 32.22%, and 34.42%, respectively, of total accumulated K during respective stages. Integration of MC with NPK proved superiority over NPK. However, the percent increase with reduced NPK level was higher (25.02% and 32.39% during tillering and grand growth phase, respectively) compared to recommended NPK (11.45% and 15.39% at the harvest and tillering stages, respectively) with the application of MC. About 61.78% higher K accumulations at the harvest stage was recorded with NPK + MC as compared to control (N_0_P_0_K_0_). Thus NPK application and inoculation of MC showed the importance of K nutrition in sugarcane crops.

### Growth, yield attributes, sugarcane and sugar yields

Application of NPK with microbial consortium improved individual cane length, diameter, and weight. The higher number of millable canes was recorded with NPK + MC compared to reduced NPK/N_0_P_0_K_0_. About 180.95% higher mean length of cane was recorded with NPK + MC as compared to N_0_P_0_K_0_. However, MC's integration increased mean cane length by 8.99% at the 50% NPK level. NPK + MC recorded a 16.12% improvement in cane diameter and 87.67% in individual cane weight over N_0_P_0_K_0_. NPK + MC also increased the number of millable canes (91,440 ha^−1^) by 23.07% over N_0_P_0_K_0_ (74,300 ha^−1^). These canes also recorded higher length and improved weight. The higher individual cane weight and number of millable canes improved cane yield (110.1 t ha^−1^) with NPK + MC. Inoculation of microbial consortium with NPK increased sugarcane yield by 21% (110.1 t ha^−1^) over recommended NPK (91.4 t ha^−1^). Reduction in NPK to half significantly affected sugarcane yield (76.04 t ha^−1^). However, the integration of MC with reduced NPK improved cane yield (95.3 t ha^−1^) by 25.33% over N_75_P_13_K_25_. Thus, concerning sugarcane yield, integration of MC with reduced NPK level (95.3t ha^−1^) showed superiority over recommended NPK (91.4 t ha^−1^). Commercial cane sugar was the product of sucrose % juice and sugarcane yield. Sucrose % juice (19.16) could also be significantly influenced by NPK + MC over N_0_P_0_K_0_ (18.34). Commercial cane sugar also followed a similar trend, as shown by sugarcane yield. Thus about 24.08% improvement in CCS was achieved by the inoculation of MC with recommended NPK. However, with reduced NPK levels, also, the increase was recorded to 28.1% over N_75_P_13_K_25_. Inoculation of MC with N_75_P_13_K_25_ could improve CCS by 10.17% (12.67 t ha^−1^) over N_150_P_26_K_50_. Thus application of microbial consortium saved 50% NPK (N_75_P_13_K_25_) and improved sugarcane and sugar yields with N_75_P_13_K_25_. However, the application of N_150_P_26_K_50_ further improved sugarcane and CCS yields.

### Agronomic efficiency of NP & K

Agronomic efficiency (Apparent recovery of N, P, and K) was worked out in kg cane produced per kg of N, P, and K applied (Table 7). N's agronomic efficiency could be increased with the inclusion of microbial consortium and inorganic sources of fertilizers. Reduction in N at 75 kg ha^−1^ produced 220.5 kg cane per kg N applied. However, increasing N dose to 150 kg ha^−1^ marginally decreased agronomic efficiency to 212.7 kg cane per kg N applied. The inclusion of MC with 50% reduced N increased it to 477.3 kg cane per kg N used. However, with the application of 150 kg N (RD), it could be reduced (337.4 kg cane per kg N applied). Recommended N with microbial consortium showed 116.5% improvement over no N and 58.63% improvement over recommended N (150 kg N ha^-1^).

**Table 7:** Growth, yield attributes, sugarcane and sugar yields as influenced by different treatments.

Treatment	Cane length (cm)	Diameter	Cane weight	NMC(000/ha)	Pol (%) Juice	Cane yield t/ha	CCS(t/ha)	AE( kg cane produced per kg N applied)	AE( kg cane produced per kg P applied)	AE( kg cane produced per kg K applied)
T_1_: N_0_P_0_K_o_	156.4	2.42	820.6	74.3	18.34	59.5	5.71	0	0	0
T_2_:N_75_P_13_K_25_	251.67	2.49	1010.00	82.71	18.38	76.04	9.89	220.5	1272.3	661.6
T_3_:N_150_P_26_K_50_	271.00	2.59	1260.00	87.40	18.31	91.4	11.50	212.7	1226.9	638.0
T_4_:N_75_P_13_K_25_ + MC	274.3	2.67	1320	89.3	18.34	95.3	12.67	477.3	2753.8	1432.0
T_5_:N_150_P_26_K_50_ + MC	283.00	2.81	1540.00	91.44	19.61	110.11	15.27	337.4	1946.5	1012.2
CD for years	29.94	0.32	144.07	10.30	2.26	10.483	1.335			
CD for treatments	14.29	0.16	92.5	3.45	0.73	6.20	1.21			
CD for years × treatments	NS	NS	NS	0.82	0.10	NS	NS			

Agronomic efficiency of P (AE_P_) was higher than N. The highest AE_P_ (2753.8 kg cane/kg P applied) was recorded with P_13_ + MC. The agronomic efficiency of P with full P (26 kg ha^−1^) + MC was higher (1946.5 kg cane per kg P applied) as compared to a similar P level without MC (1226.9 kg cane per kg P applied). This showed that the agronomic efficiency of P could be increased with the integration of MC and inorganic fertilizer. Agronomic efficiency of K ranged from 661.16 kg cane per kg K applied to 1432.0 kg cane/kg K applied in various treatments. Recommended K level (K_50_) reduced AE_K_ to 638.0 kg cane/kg K applied as compared to K_25_ (661.6 kg cane/kg K applied). However, the response at both levels could be increased with the inclusion of microbial consortium. Reduction at a higher level of K was due to a decreasing rate of increase beyond K_25_. Although sugarcane yield significantly increased up to N_150_P_26_K_50_ and at this level, the inclusion of microbial consortium further improved the yield sugarcane and sugar yields.

## Discussion

At a higher level of NPK, the inclusion of microbial consortium improved soil organic carbon by improving biological activities and decomposing crop residues containing lignin, hemicellulose, and cellulosic materials^7–10^. The mean increase in soil organic carbon during the grand growth phase was the primary source of higher degree of microbial activity at higher temperature and moisture during monsoon/rainy season^45^. NPK application increased tiller population, and during the grand growth phase, higher mortality of tillers also increased SOC at higher levels of NPK + MC. Soil microbial biomass carbon (SMBC), soil microbial biomass nitrogen (SMBN), and soil respiration (SR) are mainly governed by microbial population^46–48^ and activities. Higher rates of NPK also imparted higher soil enzymatic activity^49,50^. Soil enzyme activity is controlled by microbial population^51^. The higher population improved soil enzyme activity resulting in the degradation of complex compounds such as protein, phospholipids, lignin & cellulose significantly at a higher level of NPK and inclusion of microbial consortium.

Higher SOC leads to improved soil microbial biomass carbon^52^. Higher application of NPK and MC also made available higher N to crop and affected soil microbial biomass nitrogen^53^. Soil respiration is governed by bacterial/microbial population and represents the life of soil^54^. Higher soil organic carbon and SMBC also improved soil respiration. Residue decomposition due to increased microbial community during the tillering and grand growth phase also improved soil respiration under N_150_P_26_K_50_ and MC. Significant improvement of SMBC during the grand growth phase was recorded due to the higher microbial population in the congenial environment, i.e., rainy season-high temperature and high humidity^55^. Thus the availability of nutrients was improved in the rhizosphere that could be made available to sugarcane crops without an external supply of nutrients during the grand growth phase.

The microbial population increased due to the congenial rhizospheric environment during the grand growth phase (elongation phase) improved at the recommended level of NPK. Inoculation of *Trichoderma, Gluconacetobacter*, and *Pseudomonas* in microbial consortium further improved the soil microbial activities. *Trichoderma harzianum* induced several enzymes and inhibited soil pathogens^19,22^. *Pseudomonas fluorescens* solubilized unavailable P and produced organic acids^15,16^. *Gluconacetobacter* also makes Indole 3- Acetic Acid^12,14^. Thus congenial conditions for microbial activities were created. Improved activities of microbes also increased SMBC, SMBN, and SR at all the growth stages.

The available nutrients (NPK) status of soil was improved due to the application of NPK^56^. Further, the addition of microbial consortium enhanced nutrients availability during all the growth stages. THE mean N level increased during the grand growth phase as compared to the tillering and maturity phase. The decomposition of organic residues, higher microbial activities, and improved SMBC and SMBN also positively influenced N availability during the grand growth phase. The highest P availability was recorded during the tillering stage. The marginal decrease in P was recorded during the grand growth phase, and further availability of P increased till maturity. P fixation and availability are greatly influenced by atmospheric temperature and moisture contents of the soil. The application of P fertilizer along with PSB in MC increased P availability during the tillering phase^15^. However, initial uptake of P was found higher, so a marginal decrease in P availability in soil during the grand growth phase occurred. During the grand growth phase, loss of P due to leaching and erosion because of monsoon season might decrease P content in soil, and after passing monsoon season, the P accumulation increased during winter season^57^.

The availability of K in soil was reduced as crop growth advanced towards maturity. The amount of removal of K was higher as compared to nitrogen and phosphorus in the sugarcane crop. During the maturity phase, higher nutrients uptake from crop depleted the availability of K in soil. Higher application of K through inorganic fertilizers increased K availability in soil^56^. However, experimental soil (alluvial soil) was dominant in illite clay mineral, and the release of non-exchangeable K and availability of exchangeable K was largely dependent on K concentration in soil solution and the presence of NH_4_^+^^58^. Microbial inoculation regulates soil C and N turnover, cellulose degradation, fixes atmospheric dinitrogen, releases organic acids, influences soil enzyme activity and release of nutrients uptake and availability in soil^7,8,12,15^. In vitro activity of P solubilizers could be observed by protein production, organic acid binders, and organic P mobilization, probably due to the production of phytate^59,60^.

Cation exchange capacity (CEC) characterizes the number of fixed negative charges of plant cell walls and is an important parameter in studies dealing with the uptake of ions into roots. However, the cation exchange capacity of soil represents the capacity to hold the exchangeable cations on the clay complex. It influences the soil's ability to hold onto essential nutrients and provides a buffer against soil acidification. The higher rates of application of fertilizers released a higher number of exchangeable cations on clay micelle. Microbial population improves the soil structure and binds the particles through fungal hyphae^61^. Thus improved chemical and biological conditions affect the cation exchange capacity of soil and roots. In our experiment, the cation exchange capacity of roots was higher during the tillering stage as compared to grand growth and harvest stages. Efficiency of roots and improved cation levels due to fertilization and inoculation of MC improved CEC of roots during the tillering period^62^. Cation exchange capacity of roots was greatly reduced in control plots (N_0_P_0_K_0_) because of the poor availability of cations, particularly K^+^, Ca^++^, Mg^++^. Poor availability of exchangeable cations also affected the population of anions H_2_PO_4_^-^, HPO_4_^–^ and NO_3_^-^. When clay particles are negatively charged, they attract and hold on to cations stopping them from being leached down the soil profile^63^. This created a deficit of N, P, and K in the rhizosphere and decreased root CEC. The CEC of soil increased during the grand growth phase and decreased after that till maturity. The higher availability of nutrients during the grand growth phase, higher microbial communities and their activities, and congenial environment for grand growth improved the CEC of soil also^62^. After completion of the grand growth phase, the demand for nutrients was diminished due to sugar accumulation during the maturity of the crop. Lack of fertilization and drying of active roots in canopy reduced CEC of soil in the later phase of the crop. Improved CEC of soil due to fertilization and the microbial consortium was observed due to a higher amount of available cations and root activities.

The population of bacteria, fungi, and actinomycetes was influenced by nutrients application and microbial inoculation^64^. Application of *Trichoderma* influenced the activity and releases organic acids binding agents through hyphae and decomposed cellulose compounds^19,20^ at faster rates. *Gluconacetobacter* is IAA producing bacteria, and as endophytic bacteria, fixes N in sugarcane crop^11^. Rhizodepositions also favoured mineralization processes^65^. This increased mineralization was governed by nitrobacteria. P solubilization was also improved by the bacterial population^15,16^. The addition of NPK fertilizers also released inorganic acids controlled by microbes in soil^53^. An increased population of bacteria during the grand growth phase improved the availability of nutrients and their removal through faster residue decomposition. Population of fungi also increased during the grand growth phase. It was due to higher moisture (high humidity) and congenial temperature for fungi^66^. However, a marginal increase in the fungi population during maturity was recorded due to closed canopy, less interception of light on soil, and low temperature during maturity phase of the crop. *Actinomycetes* inhabit the rhizosphere of agricultural crops, where they increase soil fertility through the recycling of organic matter and solubilizing phosphate. The population of actinomycetes was the lowest as compared to bacteria and fungi; however, an increasing trend was observed till harvest. Slightly saline pH of soil favoured population builds up of actinomycetes^67,68^.

The tillering pattern in sugarcane showed an increasing trend till the month of June. It was due to high temperatures along with low humidity^69^. After that, due to the onset of monsoon, crop entered the elongation phase, and the tiller population decreased due to the mortality of tillers. Tiller mortality continued till September, and in the month of October, millable canes were counted. Sugarcane tillering was greatly influenced by NPK application because of the role of these nutrients in chlorophyll formation, protein metabolism^21,70,71^, root growth^72–74^ etc. However, as a growth promoter *Trichoderma and Gluconacetobacter* also favoured tiller population^13,19^. *Pseudomonas* increases P availability due to the decomposition of organic phosphorus compounds viz., phytin, phospholipids, and nucleic acids, and higher P availability improved root system^15^ of sugarcane crop. The vigorous root system due to the integration of MC with NPK also promoted tiller production in sugarcane.

Dry matter accumulation was the product of a higher number of tillers and increased leaf area. Role of N in chlorophyll formation, carbohydrate metabolism, protein metabolism, phosphorus in increasing availability of P, root growth, and K in translocation of carbohydrate^21,72,75^ improved dry matter accumulation in sugarcane. Diversion of dry matter in stalk through leaf was recorded due to regulated nutrient supply with the integration of MC. Improved physical, chemical, and biological properties of soil also regulated the supply of nutrients provided through chemical fertilizers^76^. Dry matter accumulation during the grand growth phase was higher in stalk as compared to leaf. Diversion of photosynthates from leaf to stalk and deposition of sucrose in parenchyma tissues during maturity of sugarcane crop increased dry matter of stalk. Besides, tiller mortality during the grand growth phase and stabilizing maximum leaf area during the grand growth period also decreased the contribution of dry matter in leaf. The application of MC with chemical fertilizer increased dry matter accumulation. Thus, being a high biomass producer, the sugarcane crop had great potential in increasing crop yield. Maximization in cane yield could be achieved with the integrated use of various sources for efficient nutrient supply in a balanced proportion. Higher biomass could be achieved due to improvement in soil physical, chemical, and biological conditions, higher nutrients absorption, and improved physiological and biochemical processes through the application of microbial consortium with chemical fertilizers.

Root weight and Volume were greatly influenced by nutrients application with microbial consortium. Application of NPK increased fresh root weight and Volume. Root growth and Volume increased up to the grand growth phase. However, during maturity, the root weight and volume diminished^77^. Initially, the more adventitious roots developed, and when the crop attained the grand growth phase, roots started decaying in the soil. It was also due to interculturing, earthing-up, and crop management practices. However, the weight and Volume of active roots increased up to the grand growth phase. Percent increase in root weight due to NPK application, and MC was greater during the tillering stage compared to the harvest stage. This signified contribution of plant nutrition during the early phase of crop in sugarcane. N is applied for increasing tiller emergence in sugarcane crop. During the grand growth phase, a favourable rhizospheric environment supports the growth of earlier formed tillers. These tillers receive maximum light interception, nutrients supply for their growth. Thus these tillers are converted into millable canes of higher length and weight. Increased tiller population was also recorded due to the application of MC. Higher weight and Volume of roots supported early and vigorous tillers. Microbial consortium improved root weight and Volume at both the levels of NPK. However, higher percent increase in the Volume of roots was observed as compared to fresh weight. This was due to more number of fine roots having lesser comparative weight per unit area. This affected nutrient absorption in a positive manner. Also, MC supported the growth of active roots having comparatively higher lower dry weight and higher volume^78,79^.

Nutrients uptake (NPK) was also affected by dry matter production and nutrients composition. The higher biomass was accumulated in NPK + MC plots, and these plots also removed higher nutrients per unit area. However, the differences in nutrients uptake patterns among N, P, and K were also obtained. The higher nutrient contents during tillering as compared to grand growth and maturity brought forth variations in nutrient accumulation patterns as compared to dry matter accumulation. During the tillering stage, about 38.8% dry matter production of total at harvest was recorded. However, N uptake during the tillering was about 44%. Similarly, P & K uptake also contributed 29.55% and 54% during the tillering stage as compared to the total accumulation of these nutrients at the harvest stage. This signified the application of full P and K as basal and splitting of N up to the tillering phase. Higher demand for P & K by the crop during the tillering phase improved higher rates of accumulation. Application of NPK significantly affected Chlorophyll formation, carbohydrate metabolism**,** protein metabolism, and influenced dry matter accumulation and nutrients uptake in sugarcane crop^21,23^. Besides, the role of *Gluconacetobacter diazotrophicus*^12^, *Pseudomonas*^15^, and *Trichoderma*^18^ in producing organic acids, nutrients availability, soil enzyme activities^50^, containing soil pathogens^19^, decomposing cellulosic materials^80^ and improving mineralization processes^65^ improved nutrients uptake in crop.

Effect on increasing tiller population affected the number of millable canes. The role of NPK in plant physiological and biochemical processes improved cane length having higher individual cane diameter. Cane weight was the product of individual cane length and its diameter. Thus the improvement in cane weight and number of millable canes per unit area affected sugarcane yield. Inclusion of microbial consortium at a higher rate of NPK application favoured growth, yield attributes, and sugarcane yield. The carbohydrate, protein, and fat metabolisms in plants are governed by NPK application and resulted in improvement of growth, yield attributes, cane, and sugar yields^70,71,81^. Commercial cane sugar increased due to higher sugarcane yield and improved sucrose content through the inoculation of microbial consortium with NPK. About 24.08% higher CCS (14.27 t ha^−1^) was recorded with NPK + MC as compared to sole NPK. Although, NPK + MC produced 21% higher sugarcane yield with a similar level and clearly showed the contribution of sucrose content in sugar yield (CCS) besides cane yield. Effect of microbial consortium on tillering, millable canes, growth, and yield attributes and sucrose content also brought forth improvement in sugarcane and sugar yields.

The highest agronomic efficiency of P was recorded as compared to N and K. It was due to reduced application of P (13 and 26 kg ha^−1^) as compared to N (75 and 150 kg ha^−1^) or K (25 and 50 kg ha^−1^). However, AE_N_ decreased to 212.7 kg cane/kg N applied with increasing N level from 75 to 150 kg ha^−1^. This indicated that although increasing N levels improved sugarcane yield after 75 kg N ha^−1^, however, the rate of increase was decreased. At the reduced level of N also, AE_N_ increased and proved the superiority of integration of MC with chemical fertilizers (N) as compared to sole N. At the recommended level of NPK, however, further N, P, K use efficiencies (AE) reduced as compared to 50% (NPK) + MC despite higher yields obtained at recommended NPK. Agronomic efficiency of N, P, and K were found in order of AE_P_ > AE_K_ > AE_N_. Lower doses of P and K were required to produce one kg of sugarcane as compared to N. Improved utilization of P & K by sugarcane crop as compared to N reflected higher AE of P & K than N. Higher mobility of nitrogen in soil and losses through leaching, runoff and volatilization^82,83^ decreased agronomic efficiency of N (AE_N_)_._ Thus additional N application was required to compensate for the productivity.

## Conclusions

Inoculation of microbial consortium containing new strains of *Pseudomonas fluorescens*, *Gluconacetobacter diazotrophicus* and *Trichoderma harzianum* with NPK fertilizers increased soil organic carbon as compared to inorganic fertilizers. However, soil microbial biomass carbon at the grand growth phase was increased by 137% over the control. Soil respiration with recommended NPK + microbial consortium also increased by 60% as compared to N_0_P_0_K_0._ Increasing NPK level and integration of microbial consortium improved cation exchange capacity of roots and soil at all the growth stages. The population of microbes was increased due to the application of microbial consortium. Reduction in NPK and integration with microbial consortium increased 11.16% higher biomass accumulation in stalk at the harvest stage as compared to recommended NPK. Microbial consortium alone contributed higher root biomass in the range of 26.62% (at the harvest stage) to 62.87% (at tillering stage). About 15.44% higher N accumulation in stalk at the harvest stage could be analyzed due to the inclusion of microbial consortium with recommended NPK. Reduced NPK with inoculation of microbial consortium improved about 9.90% higher P uptake in stalk than N_150_P_26_K_50_ (24.35 kg ha^−1^). About 61.78% higher K accumulation at the harvest stage was recorded with NPK + microbial consortium as compared to control (N_0_P_0_K_0_). The higher individual cane weight and number of millable canes improved cane yield (110.1 t ha^−1^) with NPK + microbial consortium. Inoculation of microbial consortium with NPK increased sugarcane yield by 21% (110.1 t ha^−1^) over the recommended NPK (91.4 t ha^−1^). About 24.08% of improvements in commercial cane sugar was achieved by microbial inoculation with recommended NPK.

Thus it could be concluded that N_150_P_26_K_50_ with microbial consortium containing *Trichoderma harzianum, Gluconacetobacter diazotrophicus, and Pseudomonas fluorescens* can sustain soil quality parameters besides improving sugarcane and sugar yields in subtropical India.

## Data Availability

Authors are ready to share the whole data of the study to Scientific Reports after publication.
